# A Simple Microfluidic Platform for Long-Term Analysis and Continuous Dual-Imaging Detection of T-Cell Secreted IFN-γ and IL-2 on Antibody-Based Biochip

**DOI:** 10.3390/bios5040750

**Published:** 2015-12-04

**Authors:** Dieudonné R. Baganizi, Loïc Leroy, Loïc Laplatine, Stacie J. Fairley, Samuel Heidmann, Samia Menad, Thierry Livache, Patrice N. Marche, Yoann Roupioz

**Affiliations:** 1 University of Grenoble Alpes, INAC-SPRAM, Grenoble F-38000, France; E-Mails: drbaganizi@gmail.com (D.R.B.); loic.leroy@ujf-grenoble.fr (L.L.); loic.laplatine@gmail.com (L.L.); sjfairley@hotmail.com (S.J.F.); samuel.heidmann@cea.fr (S.H.); samia.menad@cea.fr (S.M.); thierry.livache@cea.fr (T.L.); 2 CEA, INAC-SPrAM, Grenoble F-38000, France; 3 CNRS, INAC-SPrAM, Grenoble F-38000, France; 4 University Grenoble Alpes, Institut Albert Bonniot, Grenoble F-38000, France; E-Mail: marchep@ujf-grenoble.fr; 5 INSERM U823, Grenoble F-38000, France

**Keywords:** Cytokine, T-Lymphocytes, biochip, microfluidics, antibody, PDMS, self-assembled monolayers, SPR imaging

## Abstract

The identification and characterization, at the cellular level, of cytokine productions present a high interest for both fundamental research and clinical studies. However, the majority of techniques currently available (ELISA, ELISpot, flow cytometry, *etc.*) have several shortcomings including, notably, the assessment of several cytokines in relation to individual secreting cells and the monitoring of living cell responses for a long incubation time. In the present work, we describe a system composed of a microfluidic platform coupled with an antibody microarray chip for continuous SPR imaging and immunofluorescence analysis of cytokines (IL-2 and IFN-γ) secreted by T-Lymphocytes, specifically, and stably captured on the biochip under flow upon continued long-term on-chip culture (more than 24 h).

## 1. Introduction

The immune system is an extremely complex network involving several cell types which communicate with each other to perform specific functions in response to a multitude of cell-mediated and extracellular signals [1]. Cytokines—low molecular weight and soluble polypeptides—are major players in these networks [2]. Therefore, the identification and characterization of functional cell subsets combined with their cytokine productions are essential for both fundamental research and clinical studies. Currently, several bioanalytical assays are available for studying the immune cell activities and assessing the cytokine productions. They include, for the most common ones: flow cytometry (FC) coupled with intracellular cytokine staining (ICS) [3,4], immunohistochemistry and intracellular cytokine staining (ICCS) [5], enzyme linked immunosorbent assay (ELISA) [6,7], enzyme-linked immunospot (ELISpot) [8] and its variant—enzyme linked immunosorbent spot (Fluorospot) [9], to count cells secreting cytokines, and reverse-transcription quantitative PCR (RT-qPCR) to measure cytokine gene expressions [7,10]. Using these techniques to characterize a sample of cells, several parameters can be assessed such as the expression of cell markers (CD for cluster of differentiation) which define phenotypes of cell subsets in heterogeneous populations and the production of cytokines on a single cell basis (FC and ICCS) [3,4,5]; the extracellular secretion of cytokines in the bulk medium (ELISA) [6,7] or at the individual cell level (ELISpot) [8]. However, among many others, there are significant limitations, particularly (1) the fact that the data are collected at a single time point at the end of the analysis; that does not permit the dynamic monitoring of cell responses; (2) a high number of cells requirements to process a single assay; and (3) differences in experimental protocols such as time of collection (the snapshot in time when cell-secreted samples are collected) and the assay procedure (the method and time used for the collection of the samples) which could influence the data [3,7,11,12,13].

Microfabrication and surface engineering have provided a solution that helps to overcome most of these limitations (*i.e.*, biosensor or biochip approaches), thus allowing having a better indication of the cell activity *in vivo* [14,15,16]. Numerous studies on the development of micropatterned surfaces for the analysis of cytokine-secreting immune cells are documented in scientific literature [14,15,16,17,18]. Chen *et al.* [19], for instance, applied reconfigurable microfluidics with an antibody array containing antibody probes specific to cell-surface antigens, as well as cytokine detection probes. This microarray allowed to capture specific leukocytes (CD4+ T lymphocytes) and, thereafter, to detect their secreted cytokines (IL-2, TNF-α, and IFN-γ). Moreover, other studies went a step further by developing cellular microarrays for continuous monitoring of cytokine secretion [20,21,22]. For instance, Liu *et al.* [20] described an aptasensor array using aptamer-modified electrodes packaged in a non-fouling hydrogel and integrated with microfluidics. This array enabled the capture of CD4+ T cells from a human leukocyte sample and continuous detection of IFN-γ release from these cells [20]. Likewise, some studies have, in turn, coupled cellular microarrays with Surface Plasmon Resonance Imaging (SPRi), an optical technique allowing label-free and real-time analysis commonly used to detect specific interactions between different molecules [23,24,25,26]. In doing so, Milgram *et al.* [26] described an antibody microarray using SPRi detection to simultaneously monitor in real-time T cell secretions of both IFN-γ and IL-2 after cellular mitogenic stimulation. The biochip was composed of microarrayed antibodies specific to T lymphocytes (anti-CD3e, anti-CD28), antibodies specific to secreted cytokines (anti-IFN-γ, anti-IL-2), and with a combination of both antibodies. Bindings of cells and/or cytokines with grafted probes were detected by SPRi. Although these systems provide promising advances, the cell secretions of cytokines are detected over a short incubation period, typically after 1–6 h of cell incubation on the biochip [18,19,20,21,25,26]. Since the kinetics of cytokine synthesis and secretion differ among cytokines [12,13,25,27], the analysis of cytokines on a short incubation time limits the ability of these techniques to perform functional analysis of cells according to cytokine secretions. This applies in particular to studies aiming to provide a complete characterization of functional modulations which can arise from certain cell cycle phases, varying half-life of secreted cytokines, and the variations regulation of cytokine productions.

In the present work, based on the aforementioned biochip analysis format previously described [26,28], we have developed a system capable of handling the monitoring of cytokine secretions upon continued long-term cell culture of viable T lymphocytes. To achieve this goal, a strategy based on T cell-specific and cytokine-specific antibodies was used: T cells injected on the biochip via a fluidic system were first captured on the microarray surface through specific antibodies recognizing their differentiation markers and afterwards, the cytokines secreted by captured T cells were detected by interaction with their specific antibodies grafted in close vicinity of secreting cells. Cytokine secretions were recorded either by fluorescence labeling with a specific antibody after 24 h of on-chip cell culture or by direct measurement of SPRi signals for up to 65 h.

## 2. Experimental Section

### 2.1. Materials and Reagents

Phosphate buffer saline (PBS), phorbol 12-myristate 13-acetate (PMA), phytohemagglutinin (PHA), ionomycin, trypan blue, absolute ethanol, sodium dihydrogen phosphate (NaH_2_PO_4_), sodium phosphate dibasic (Na_2_HPO_4_), sodium hydroxide (NaOH), sydrochloric acid solution (1N HCl), paraformaldehyde A (PFA), and glass slides coated with transparent and electrically-conducting film of indium-tin-oxide (ITO), were purchased from Sigma–Aldrich (St. Quentin-Fallavier, France); whereas N-2-hydroxyethylpiperazine-N-2-ethane sulfonic acid (HEPES), AIM-V Serum Free Medium, and Dulbecco’s 1X PBS without calcium and magnesium were purchased from GIBCO^®^ (Cergy Pontoise, France). Human ELISA IFN-γ and Human ELISA IL-2 Ready-Set-Go (10 × 96 Tests) protocol kits were purchased from eBiosciences (Rennes, France), LIVE/DEAD^®^ Cell Vitality Assay Kit and dimethyl sulfoxide (DMSO) from Molecular Probes (Cailloux-sur-Fontaines, France‎), Vivaspin filter membranes from Vivascience (Palaiseau, France), Thiol-PEG: HS-C_6_-(CH_2_-CH_2_-O)_6_-OH; from Prochimia (Sopot, Poland), PDMS and its curing agents Sylgard 184 from Dow Corning (Seneffe, Belgium), and thermal paste (Silicone Heat Release Transfer Compound Thermal Paste—White) from DX DealExtreme (Paris, France). Human monoclonal antibodies used for the capture of T lymphocytes and cytokines consisted of the following: purified anti-CD3 (clone UCHT1), anti-CD19 (clone HIB 19), anti-Interferon gamma (clone NIB42), anti-interleukine 2 (clone MQ1-17H12), and Mouse IgG (IgG1κ isotypic control) were all supplied by eBiosciences (Rennes, France). Antibodies used for cytokine immunosandwich detection are listed below: polyclonal Anti-Human IL-2 Biotin (1 mg·mL^−1^) and monoclonal Anti-Human IFN-ɤ Biotin (clone 4S.B3; 1 mg·mL^−1^) were also purchased from eBiosciences (Rennes, France).

### 2.2. Cell Sample

In this work, primary T lymphocytes isolated from the whole blood of healthy adult donors supplied by the *Etablissement Français du Sang* (EFS, Grenoble-France) were used. They were purified using standard Ficoll-Paque PLUS gradient centrifugation (GE Healthcare Life Sciences, Velizy-Villacoublay, France) and subsequently isolated using a Human Pan T cell isolation kit (Miltenyi Biotec, Paris, France) following the manufacturer’s instructions. The freshly isolated peripheral blood T lymphocytes were washed with Dulbecco’s 1X PBS; re-suspended in AIM-V Serum Free Medium buffered to the pH of 7.2–7.4 range with 25 mM HEPES and immediately used. Prior to use on the biochip experiments, they have been characterized by fluorescence-activated cell sorting (FACS analysis) using the LSRII flow cytometry apparatus (BD Biosciences) equipped with BD FACSDiva6 software to confirm the expression of T cell surface differentiation marker CD3 and to evaluate the activation efficiency by measuring the expression of surface marker CD69. For this purpose, cells were suspended in 100 µL of Dulbecco’s PBS 1X at a concentration of 1 × 10^6^ cells. Afterwards, they were incubated with 5 μL of each antibody for 15 min at 4 °C in the dark. They were finally washed with Dulbecco’s PBS 1X (centrifugation at 800 g for 7 min) and re-suspended in 500 μL of Dulbecco’s PBS 1X for FACS analysis. FACS data were further analyzed using FCS Express V4 software. Their secreting activity was also evaluated by ELISA assay in culture supernatant. Nearly all (>95%) were CD3 positives and CD19 negatives, and after activation with PMA (100 µg·mL^−1^) associated with ionomycin (500 µg·mL^−1^), more than 95% of CD3+ were activated (measured by flow cytometry detection of CD69 expression). Prior to the injection, cellular samples (cell viability >95%, evaluated by trypan blue staining) were re-suspended at a cell concentration of 10^6^ cells per 100 µL in fresh culture medium containing stimulants.

### 2.3. Biochip Preparation and Surface Treatment

**Figure 1:**
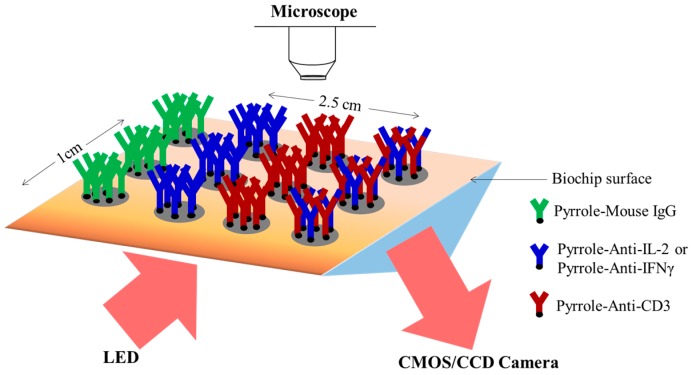
General scheme describing the design of the biochip for the capture of T-cell and the detection of secreted cytokines. Pyrrole-conjugated cell- and cytokine-specific antibodies (Anti-CD3, anti-IL-2 or anti-IFN-γ, and Mouse IgG as negative isotypic control) are electrochemically grafted side-by-side or mixed on the biochip surface (~300–400 µm of spot diameter). The grafted biochip is subsequently treated with Thiol-PEG to prevent nonspecific adhesion of cells on unfunctionalized regions across the biochip surface.

The biochip was fabricated on a solid material based on a glass slide (Advanced Optics—Schott, Clichy, France) or a prism (Schott, Yverdon, Switzerland) bearing a 50 nm thick gold layer lying on a 1 nm thick chromium layer (1 × 2.5 cm). Prior to use, it was oxygen plasma cleaned (standard plasma system FEMTO; Electronic Diener, Ebhausen, Germany) and stored overnight at 4 °C to keep the surface wettability and avoid recontamination. The biochips were electrochemically arrayed with antibodies as described previously (Suraniti *et al.* [26]; Milgram *et al.* [28]). Briefly, antibodies (0.5 µM) were conjugated to N-hydroxysuccinimidyl 6-(pyrrolyl)-caproate; NHS-pyrrole (provided by ERAS Laboratory, Saint-Nazaire-les-Eymes, France), final concentration of 200 µM in 1X PBS pH 7.4 overnight at 4 °C. After coupling, antibodies were purified by ultracentrifugation on Vivaspin filter membranes (cutoff 30 kDa MWCO) for 15 min at 15,000 g and re-suspended in the spotting buffer (50 mM phosphate buffer, pH 6.8, containing 50 mM NaCl and 10% glycerol). Pyrrole-conjugated antibodies were then mixed with free pyrrole monomers (ratio 1:20,000) in the spotting buffer to the final concentration of 2 μM. Afterwards, they were grafted on the chip by electro-copolymerization of free pyrrole and pyrrole-modified antibodies (2V ddp, for 100 to 500 ms) [26,28]. This technique enabled the microarraying of probes of average diameter of 300–400 µm with 1 mm center-to-center spacing. Probes of T lymphocyte-specific antibody (anti-CD3), cytokine-specific antibody (anti-IL-2 and anti-IFN-γ), a mixture of anti-CD3 and anti-IL-2 or anti- IFN-γ (ratio 50:50, final concentration of the mixture equivalent to that of each antibody used alone), and mouse IgG isotypic control were grafted side by side on the biochip surface (Figure 1). Anti-CD19 was also grafted in some experiments as the control of cell capture specificity. For SPRi dual-analysis, anti-CD3, anti-IL-2, anti-IFN-γ, a mixture of anti-CD3/anti-IL-2 and anti-CD3/anti-IFN-γ, mouse IgG isotypic control alone or mixed with anti-CD3, anti-IL-2, and anti-IFN-γ were all grafted side by side on the biochip surface. All antibodies were grafted in triplicate for each experiment.

In order to prevent nonspecific adhesion of cells on the biochip surface, a non-fouling treatment of the surface with polyethylene glycol thiol self-assembled monolayers (Thiol-PEG SAMs) was performed. A stock solution of 50 mM Thiol-PEG was prepared by diluting the Thiol-PEG in absolute ethanol. The Thiol-PEG treatment was performed overnight at room temperature by spreading 50 µL of a Thiol-PEG working solution of 1 mM in an aqueous solution of 50 mM phosphate buffer (1 M NaH_2_PO_4_, and 1 M Na_2_HPO_4_ in distilled water, pH 7.5) on the biochip surface which was then covered by a coverslip and left in a humid chamber. Finally, the biochip was washed with 50 mM phosphate buffer and stored at 4 °C in 1X PBS.

### 2.4. Design of the Microfluidic Platform

**Figure 2:**
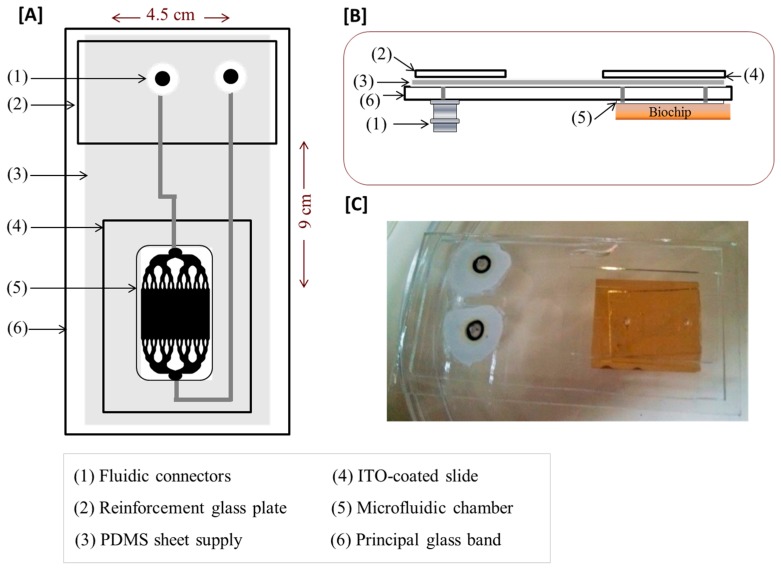
The design of the microfluidic platform integrated with the antibody-based biochip. (**A**) 2D view and presentation of different parts; (**B**) side view; and (**C**) picture of the device.

A microfluidic platform was developed in order to perform all the assay operations on a single analysis, including cells’ on-chip capture and long-term culture; and to increase their local concentration while sampling a maximum number of cells. A polydimethylsiloxane (PDMS) microfluidic chamber device was prepared by mixing the PDMS with the curing agent (ratio 10:1), poured onto a smoothed aluminum replica mold, degassed in a vacuum chamber, and cured at 70 °C overnight. In the same way, the PDMS sheet supplying of the microfluidic chamber was prepared and poured onto a SU-8 micropatterned silicon (Si) wafer mold. The active side of the PDMS microfluidic chamber was bonded on a side of a glass band (9 × 4.5 cm, 0.3 cm of thickness) where the fluid inlet and outlet were previously punched (Figure 2). The PDMS supplying of the microfluidic chamber was, in turn, bonded on the other side of the glass band. Finally, an Indium-Tin-Oxide (ITO)-coated glass slide (3.5 × 2.5 cm) was bonded to the PDMS supplying of the microfluidic chamber (above the microfluidic chamber) ensuring that the conducting side of the ITO coated glass slide remains exposed to the surface (Figure 2). Both the glass band and the PDMS structures were oxygen plasma activated for 30 seconds before bonding (Standard plasma system FEMTO; Electronic Diener, Ebhausen, Germany). The microfluidic reaction chamber was of 1 × 2.5 cm, 300 µm of thickness, 125 µm in depth, 8 × 8 mm of active surface and approximate volume of 80 µL. In these conditions, the flow was laminar and the inertial effects were considered as negligible. The range of flow applied was between 0.05 µL·s^−1^ and 1 µL·s^−1^ which corresponds to a speed below a millimeter per second in the microfluidic chamber (average linear flow rate < 1 mm·s^−1^ and Re << 1). The observed flow of the cells before attachment corresponded to a calculated velocity of ~1.5 × 10^−5^ m·s^−1^ and the constraint applied on cells (*i.e.*, wall shear stress) was between 0.015 and 0.03 Pa [29]. The assembly, which remains transparent, was compatible with any mode of optical microscopy and optical evanescent wave biosensing.

The microfluidic platform was mounted on a Leica DMI4000 B Automated Inverted Microscope (Leica Microsystems, Nanterre, France) equipped with Lumenera’s INFINITY2-2 digital CCD camera to record images and videos. For this, a specific plate adapted to the microfluidic assembly and compatible with the Leica DMI4000 B microscope has been designed and fabricated. Cells were observed in real-time with digital magnification of ∑100X either in new integrated phase contrast (IPH) and Leica’s differential interference contrast (DIC).

### 2.5. SPRi Detection Set-Up

**Figure 3:**
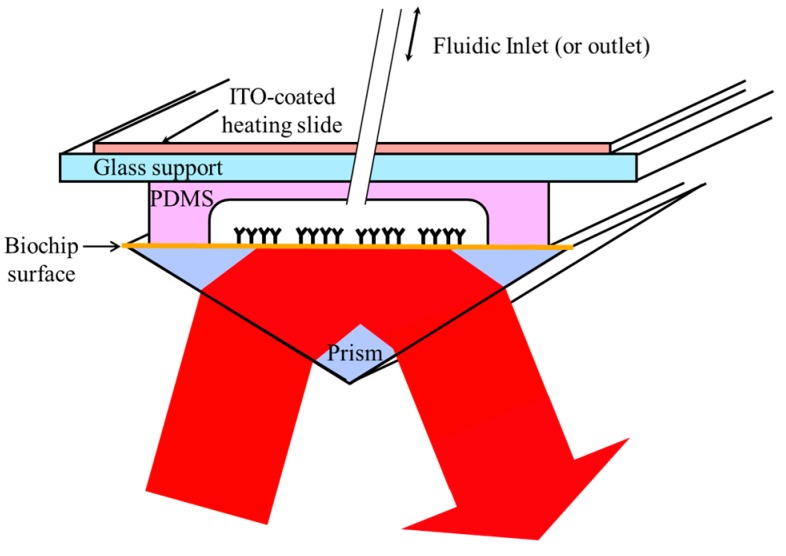
General scheme describing the SPRi set-up. The system is built in layers starting from the biochip (prism) surface at the bottom, the PDMS chamber with the fluidic inlet/outlet, the glass support, and the ITO-coated glass slide for heating and thermal regulation. A laser LED of a wavelength of 735 nm is used as light source.

In order to perform both SPRi and optical microscopy simultaneously, the microfluidic platform previously described was associated with a homemade and open SPRi setup, allowing fluidic control and optical imaging from above [30]. Briefly, the system was built in layers starting from the biochip (prism) surface at the bottom, the PDMS chamber with the fluidic inlet/outlet, the glass support, and the ITO-coated glass slide for heating and thermal regulation (Figure 3). A laser LED of a wavelength of 735 nm was used as light source. Once collimated and polarized through a grid, the light enters the prism, reflects on the back of the functionalized surface and is collected by a CMOS 16-bit camera (ORCA Flash 4.0, Hamamatsu Photonics K.K., Hamamatsu, Japan). The spatial resolution of our SPR sensor at 735 nm wavelength incident light is about 1.7 to 5 µm. The ultimate lateral resolution is 1.7 and 2.8 µm respectively perpendicular and parallel to the surface plasmon waves (SPW) at λ = 632 nm in water on field-of-views as large as 10 mm^2^.

### 2.6. Heating and Thermoregulation in the Microfluidic Cell

**Figure 4:**
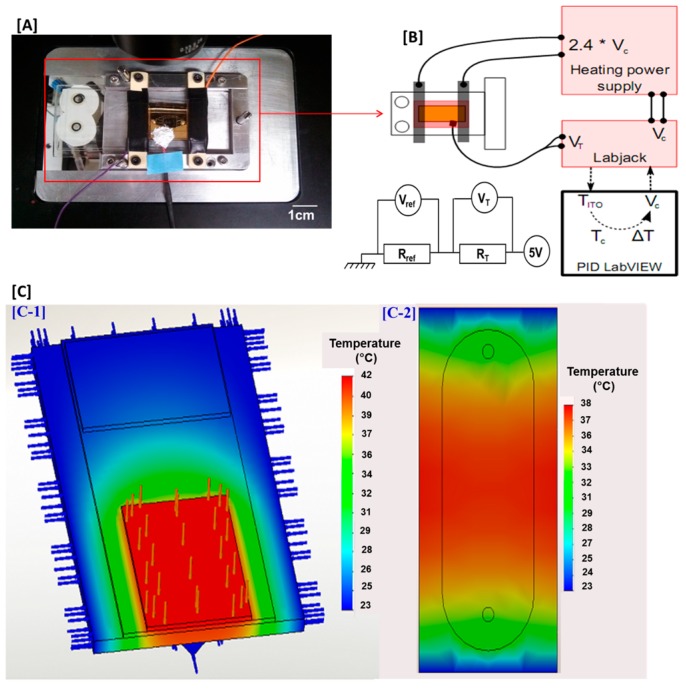
The thermal regulation device. (**A**) Picture of the experimental setup of the biochip and the PDMS microfluidic assembly mounted on a homemade Leica DMI4000 B microscope plate and connected to the thermal regulation device; (**B**) scheme of the thermal measurement and regulation system; and (**C**) stationary temperature field simulation performed on SolidWorks. The ITO-coated glass slide is heated-up at 42 °C to obtain a temperature of 37 °C in the microfluidic chamber. (C-1) Global view of the device; Temperature scale: 23 °C to 42 °C. (C-2) Field temperature on the biochip surface; Temperature scale: 23 °C to 38 °C.

Different approaches have been adopted to establish either a uniform temperature or a constant temperature gradient in a biochip, from external heating sources to Joule heating, microwaves, or the use of lasers to cite just a few examples [31,32,33]. Their main advantage is that they involved simpler and significantly less expensive manufacturing materials while allowing a faster regulation over a larger range of temperatures (from 0 °C to 120 °C) [33]. In this work, a spatially-localized heating system by Joule heating effect with a glass slide coated with transparent and electrically-conducting film of indium-tin-oxide was incorporated to the device to maintain the microfluidic chamber in a physiological environment (37 °C) (Figure 4). This model facilitates both homogeneous temperature regulation within the whole microfluidic chamber and linear temperature profiles with a high degree of accuracy. The low thermal conductivity of the PDMS vessel ensures the control and stability of a uniform temperature profile within the microfluidic cell [31]. The heat was generated by two copper electrodes placed onto the ITO-coated glass slide above the microfluidic device when an electric field was applied across the ITO-coated glass slide. Thermoregulation was done through a thermistor whose head was glued on the surface of the ITO-coated glass slide (R ~ 15 Ω) and on which some thermal paste was added to ensure good thermal conduction. The system was PC-controlled by thermoregulation LabVIEW software mainly composed of a PID controller. The PID aims to reduce the difference of temperature between the setpoint temperature and the ITO-coated glass slide’s temperature by adjusting the voltage V from 0 to 5 V at the output of the LabJack. In this setup, the temperature of the microfluidic chamber could be regulated from room temperature up to 47 °C at accuracy less than 1 °C and a precision less than 0.25 °C over more than 48 h. This system used a very low heating power (<2 W) to avoid a fast and elevated flowcell heating that could damage arrayed antibodies and cells. The ITO-coated glass slide was heated-up at 42 °C to obtain a temperature of 37 °C in the microfluidic chamber. More details are presented in the Supplementary.

### 2.7. On-Chip Cell Culture in the Microfluidic Device

The fluidic assembly was composed of a PC-controlled Cavro^®^ XLP 6000 Modular Syringe Pump (Tecan, Lyon, France) equipped with a 0.5 mL syringe, allowing to set the flow from 0.05 µL·s^−1^ to over 30 µL·s^−1^. Appropriate solutions were delivered by polyether ether ketone (PEEK) tubes (ID: 760 µm, OD:1500 µm; IDEX Health and Science, Wertheim-Mondfeld, Germany) through a two-channel degassing module (AllTech Elite™ Degassing System, Fisher Scientific, Illkirch-Graffenstaden, France) and a switchable three-channel injection valve coupled to the transparent flowcell. Additionally, the outlet of the flowcell has a switchable valve enabling to close the microfluidic chamber (together with the injection valve).

Prior to the experiments, tubing, valves and devices were first sterilized with 70% ethanol for 15 min. The system was then filled with sterile distilled water and then Dulbecco’s 1X PBS at a flow rate of 1 µL·s^−1^ to prime the device and tubing. Afterwards, the degassing module was turned off and the pre-heated AIM-V medium buffered with 25 mM HEPES and containing 100 ng·mL^−1^ PMA and 500 ng·mL^−1^ ionomycin was loaded at a flow rate of 0.5 µL·s^−1^ and the system left on for about 30 min. Cellular samples (100 µL; 10^6^ cells) were then injected. Afterwards, a system of back and forth of cells was set up at a flow rate of 0.05 µL·s^−1^ for 30 min to 1 h allowing a maximum of cells to interact with arrayed antibodies. Thereafter, the flow rate was increased to 0.5 µL·s^−1^ in order to wash away nonspecifically bound cells. The number of cells captured is ~300 cells per probe. The microfluidic chamber was then closed and the analysis was led for 24 h. Finally, the biochip was recovered from the microfluidic device for further analysis.

### 2.8. On-Chip Cell Vitality Assay

In order to ensure that on-chip captured and cultured cells remain viable and bioactive, an assay that distinguishes metabolically active cells from injured or dead cells was performed directly on chip. The LIVE/DEAD^®^ Cell Vitality Assay Kit was adapted to the on-chip analysis and optimized. 2.5 µM working solutions of C12-resazurin in DMSO and 25 nM working solution of SYTOX^®^ Green stain also in DMSO were prepared. A solution of 1% paraformaldehyde A (PFA), pH 7.4 was also prepared for cell fixation and filtered with 0.2 µm syringe filters. For this purpose, the biochip was recovered from the microfluidic chamber after 24 h-analysis and incubated for 15 min at 4 °C with 1% PFA. The slide was then washed by gentle dipping it in a solution of Dulbecco’s 1X PBS carefully to not take off cells. Afterwards, a mix of 150 μL of 2.5 µM C12-resazurin and 150 μL of 25 nM SYTOX Green in Dulbecco’s 1X PBS was applied on the biochip which was thereafter incubated for 15 min at 37 °C with 5% CO_2_. After the incubation period, 1 mL of Dulbecco’s 1X PBS was added and the biochip immediately observed with Olympus BX60 Epifluorescence Microscope equipped with a CCD camera (Olympus, Rungis, France) and Image-Pro Plus software.

### 2.9. On-Chip Cytokine Detection

The cytokine secretions were analyzed on the one hand continuously by SPRi and, on the other hand by immunofluorescence after on-chip cell capture and culture (24 h). The SPRi data were acquired at regular intervals upon cell injection and capture on their specific antibodies. Areas of interest were drawn on the biochip surface corresponding to grafted antibody probes and raw data were corrected by every probe sensitivity given by the plasmon curve. The reflectivity variations of each grafted probe triplicates were averaged and plotted upon time after rectification by the subtraction of the negative control signals (*i.e.*, mouse IgG and/or anti-CD3 mixed with mouse IgG) to eliminate the general bulk solution index shift and the SPR signal from the cells. The immunofluorescence detection of cytokines secreted and bound to their specific antibodies on the biochip was performed in an immunosandwich assay after on-chip cell capture and culture and subsequent washing away of cells. 300 µL of biotinylated anti-human IL-2 or IFN-γ diluted to 1:300 in 1X PBS were applied on the biochip for 1 hour at room temperature, followed by a profusely wash in Dulbecco’s 1X PBS and 15 min of conjugation with 100 µL of 5% streptavidin-phycoerythrin. After further washing in Dulbecco’s 1X PBS, the fluorescence was observed with an exposure time of 0.04 s and gain ×4 using Olympus BX60 Epifluorescence Microscope equipped with a CCD camera (Olympus, Rungis, France) and Image-Pro Plus software. The fluorescence image of each probe pattern was recorded and later analyzed by averaging the grey level (mean fluorescence intensity) of each pixel contained in a region of interest, matching each individual probe pattern. An area of the biochip without a probe pattern was used as control of the background noise. The area size of probe patterns was kept constant for the analysis of an entire biochip.

### 2.10. ELISA Titration of Cytokines

In order to assess the secretion of cytokines IFN-γ and IL-2, the culture medium was recovered from the microfluidic reaction chamber by pipetting (~50–80 µL) and the cytokines dissolved in the bulk medium assessed by ELISA assay. The ELISA assay was performed following the eBiosciences Ready-Set-Go protocol kit instructions. Briefly, the 96-well ELISA plates were coated with anti-IL-2 or anti-IFN-γ capture antibody overnight at 4°C, washed and blocked with specific buffers of the kit. Then samples and standards were added to the wells. Bound antibodies were revealed with streptavidin-HRP using tetramethyl benzidine (TMB) as the substrate. Standard curves were plotted using dilutions of the standard solution provided in the kit.

## 3. Results and Discussion

### 3.1. Specific Capture and Culture of Viable T Cells on the Biochip

A system that allows T cell-specific capture by using an antibody microarray chip for specific binding of cells to the biochip under flow was first developed. The biochip surface was designed and optimized to capture of T cells in an active state supporting efficient recognition of their membrane proteins and specific cytokines—detector reaction. To capture cells, the biochip was arrayed with monoclonal antibodies recognizing CD3, specifically-expressed by T lymphocytes (anti-CD3), CD19, expressed by B lymphocytes but not by T lymphocytes, as the control of cell capture specificity and with mouse IgG as negative isotypic control. For cytokine detection, specific antibodies (to IL-2 and IFN-γ), both secreted by T lymphocytes but not by B lymphocytes were arrayed [26,28]. In order to minimize non-specific adsorption of cytokines or whole cells on unfunctionalized regions across the biochip surface and to improve the reproducibility and to reduce the “background” cell binding level, a self-assembled monolayers (Thiol-PEG SAMs) treatment with HS-(CH2)_6_-(CH_2_-CH_2_-O)_6_-OH was performed.

**Figure 5:**
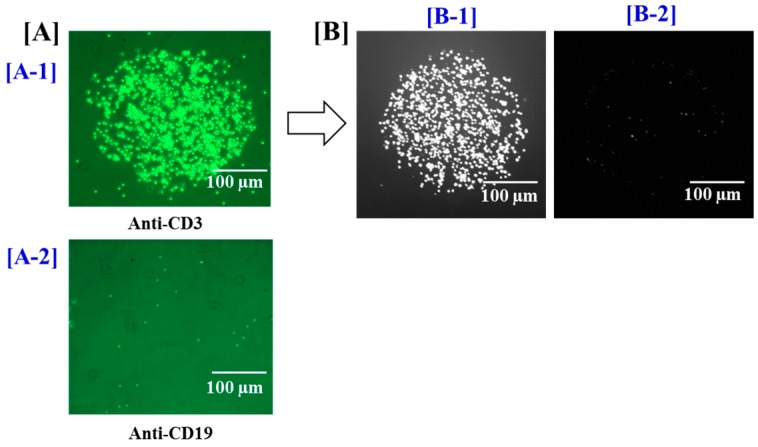
Specific capture of viable peripheral blood T lymphocytes on the chip. (**A**) Phase Contrast Microscopy images of antibody probes (objective 10× + Σ100× digital magnification): (A-1) T lymphocytes are specifically and stably bound on their specific antibody (anti-CD3); (A-2) there is no capture on negative control anti-CD19); and (**B**) fluorescence images of healthy and metabolically active captured T cells (B-1) and dead cells (B-2) after on-chip staining with cell vitality assay.

The Figure 2 illustrates the polydimethylsiloxane-based (PDMS) microfluidic platform subsequently developed for T cell on-chip capture and long-term culture. This system was built by using biocompatible materials highly adapted to cell analysis. In addition, HEPES buffer was added to the cell culture medium to maintain the physiological pH in the microfluidic chamber. The microfluidic device contained connections at the inlet and outlet of the microfluidic cell to further allow a homogenous distribution of cells in the microfluidic chamber. In order to maintain a physiological environment within the chamber, a heating system was added to the device to maintain the temperature at 37 °C. Only the temperature in the microfluidic chamber was regulated by spatially-localized heating (Figure 4). Peripheral blood T lymphocytes (10^6^ cells) suspended in 100 μL AIM-V Serum Free Medium buffered with 25 mM HEPES and containing 100 ng·mL^−1^ of PMA and 500 ng·mL^−1^ of ionomycin for cell stimulation were injected. In this biochip format, T cells were specifically and stably captured on their specific anti-CD3 antibodies. This was observed in real-time thanks to the digital CCD camera mounted on the microscope. There was very low or no unspecific capture observed on anti-CD19 antibody or on mouse IgG controls (Figure 5 and Figure 7). This correlated the relative abundance of T *versus* B cell populations as determined by flow cytometry of cell samples prior to their use, yielding to over 95% of CD3 T cells and less than 5% of CD19 B cells. Furthermore, it was important to ensure that cell capturing on the biochip did not adversely affect cell viability and that cells bound on the biochip via antibody-antigen interactions remained viable and bioactive. Using a cell vitality assay, the fluorescence intensity corresponding to healthy and metabolically active cells was emitted by over 95% of captured cells unlike the fluorescence intensity corresponding to dead cells which was not detected (Figure 5). Moreover, the majority of cells recovered from the biochip by gentle pipetting showed good viability (>90%). This clearly showed that cell viability and activity were not affected by their capture and long-term culture on the biochip.

### 3.2. Detection of Captured T Cell Cytokine Secretions

The biochip developed in this study was designed to measure cytokine secretions (IFN-γ and/or IL-2) secreted by captured T cells after cellular stimulation and on-chip culture. Moreover, it has been especially designed to be compatible with both optical microscopy and SPRi. We have focused on the cytokine secretion that occurs in cells activated by an external chemical stimulation. The *in vitro* T cell nonspecific activators were used, *i.e.*, ionophore agents; well known for their faculty to activate T cells leading to the enhancement of cytokine production: PMA (100 µg·mL^−1^) was combined with ionomycin (500 µg·mL^−1^) [34]. The biochip was designed in order to ensure the specific binding of cells in an active state (primary cultures) and cytokine specific detection in the close vicinity of the captured cells. Anti-CD3 antibodies were grafted to bind T cells, anti-IL-2, or anti-IFN-γ to bind the cytokines produced in bulk, and a mixture of either anti-CD3/anti-IL-2 and/or anti-CD3/anti-IFN-γ to detect the signal corresponding only to the cytokine secreted by captured T cells. A non-relevant mouse immunoglobulin isotypic control was used as a negative control.

**Figure 6:**
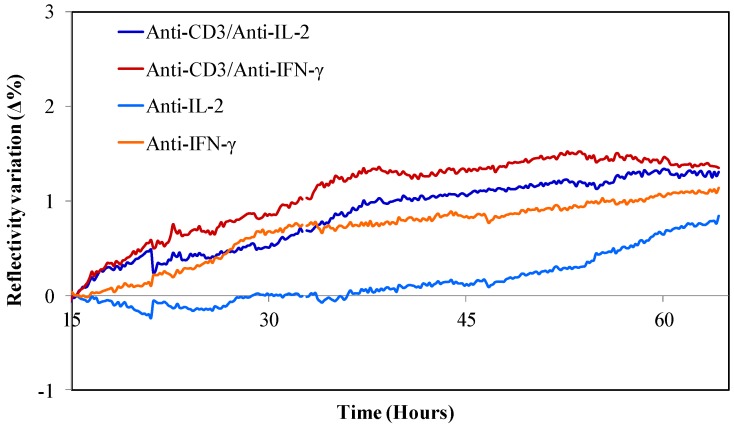
Continuous SPRi dual-detection of IFN-γ and IL-2 secreted by captured peripheral blood T lymphocytes. After cell injection in the microfluidic chamber and capture on their specific antibody probes (anti-CD3, mixture of anti-CD3/anti-IL-2, and anti-CD3/anti-IFN-γ), secreted cytokines are continuously detected by SPRi. Reflectivity variations of each probe are averaged and plotted upon time after rectification by the subtraction of the control signals (mouse IgG and/or anti-CD3 mixed with mouse IgG).

The SPRi detection was performed continuously and simultaneously for IFN-γ and IL-2 secreted by peripheral blood T lymphocytes starting after their capture on their specific antibodies and up to more than sixty hours. The main advantage of SPRi is that it allows detecting the interaction of cytokines with their specific antibodies arrayed on the biochip as refractive index variations in both label-free and real time manner [25,26]. Moreover, this technique has been shown to be highly sensitive and effective for the analysis in real-time of cell- and/or secretion-surface binding detection [23,25,26,28,35]. The SPRi real-time monitoring of cytokine secretions from a living cell population for an extended incubation time requires maintaining the cells in a physiological environment. Therefore, we have built a microfluidic platform that, as shown in Figure 5, allows long-term culture of cells on the biochip without affecting their viability and activity. The capture of cells on each arrayed features was observed in real-time by both regular bright field microscopy and SPRi. SPR images of probes were snapped at regular intervals. The reflectivity variations of the surface of grafted antibody probes were averaged for each probe triplicates. The specificity of the reaction was verified thanks to the mouse IgG control probes (mouse IgG and/or anti-CD3 mixed with mouse IgG). The plotted curves upon time after rectification by the subtraction of the control signals confirmed the highly-specific recognition and detection of IFN-γ and IL-2 secreted by the captured cells or present in the bulk (Figure 6 and Supplementary). However, the SPRi and optical microscopy observations show that at the beginning, the majority of the cells are not perfectly captured and move slightly on probes. Therefore, the relative change of SPR curves is observed from the moment that the dynamics due to cell movements is stabilized (~15 hours in our experiments). Nonetheless, when the curves are put to zero earlier, the results are not fundamentally different; therefore, this phenomenon does not strongly perturb the time monitoring of secreted cytokine.

**Figure 7:**
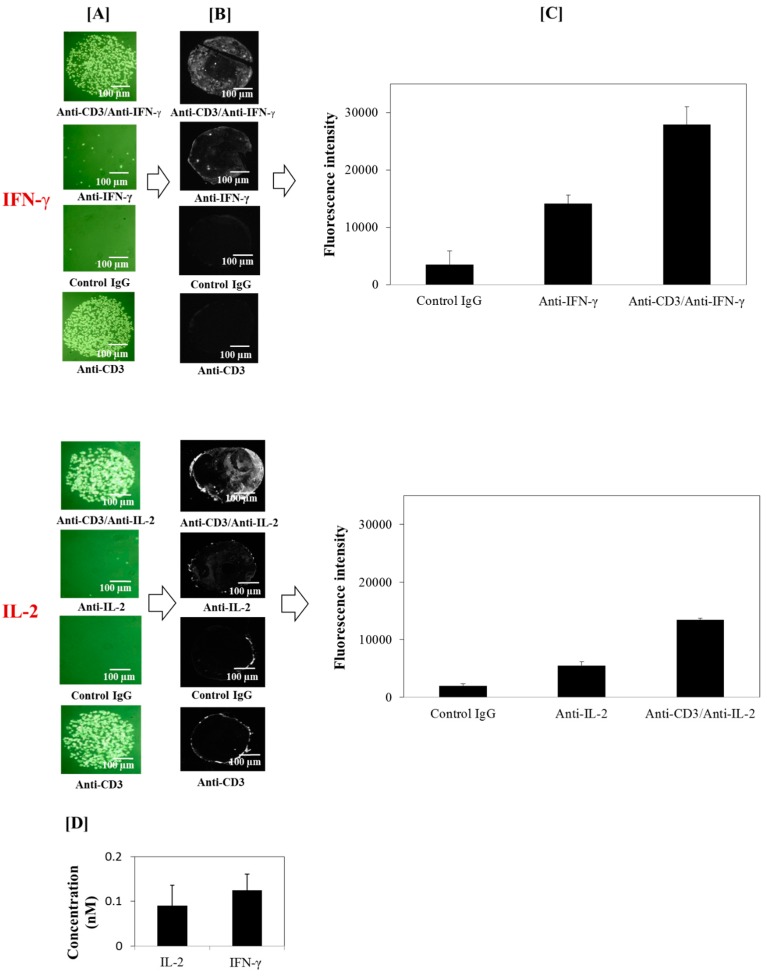
On-chip immunofluorescence detection of IFN-γ and IL-2 secreted by captured peripheral blood T lymphocytes. (**A**) Phase Contrast Microscopy images (objective 10× + Σ100× digital magnification) of lymphocytes which are specifically and stably bound on the specific anti-CD3 antibodies (alone or in combination with anti-IFN-γ or anti-IL-2 antibodies). There is very low or no unspecific capture of T cells on anti-IFN-γ, anti- IL-2, or mouse IgG isotypic controls; (**B**) fluorescence images (objective 10×, gain ×4, and exposition time 0.04 s) of secreted IFN-γ or IL-2 after detection with secondary anti-cytokine antibodies labeled with biotine and revealed by (strept)avidin-phycoerythrin; and (**C**) fluorescence intensities of secreted IFN-γ and IL-2 (*n* = 5). [**D**] ELISA titration of cytokines in cellular supernatants after on-chip cell culture (24-hours). After each experiment, the culture medium was recovered from the microfluidic reaction chamber (~50–80 µL) and the cytokines dissolved in the bulk medium measured by ELISA (*n* = 5).

The fluorescent detection involves the biotin-(strept)avidin system in an immunosandwich assay. The biotin-(strept)avidin system is one of the most widely employed detection method in biosensor applications as it exhibits highly-specific and strong binding affinity and, thus, generates intense signals and low background [36]. In this immunosandwich format, only target molecules bound to the surface are labeled by the IL-2- or IFN-γ-specific secondary antibodies giving a low background signal. The graphical representation of the fluorescence measures indicates the specific recognition between cytokine-specific antibodies and secreted cytokines detected (Figure 7). No fluorescence was observed on the mouse IgG control probes, thus assessing low background adsorption on control patterns. Furthermore, the subtraction of non-specific responses showed locally-high fluorescence intensity corresponding to the cytokines secreted by the captured cells (Figure 7). This was considered as individual cell-secreted cytokines since they were detected before the cytokines diffuse in the culture medium, while the non-captured fraction which diffused into the medium was considered to be part of the overall secreted cytokines present in the bulk. In fact, it has been shown that the cytokine would be expected to remain in the vicinity from a point source for at least 100 s [37,38,39]; which is a sufficient time for the cytokine to encounter the capture antibody. To confirm that the fluorescence signal detected corresponds to the cytokines secreted and not to artifacts, the culture medium was recovered from the microfluidic reaction chamber (~50–80 µL) and the cytokines present in the bulk medium measured by ELISA assay. In this configuration, the average concentration in the supernatant was around 0.1 nM for IL-2 and 0.15 nM for IFN-γ, thereby confirming the cytokine secretory activity of captured T cells (Figure 5). Hence, this biochip analysis system allows detecting local cytokine secretions below nanomolar concentrations in the bulk medium; levels which are undetectable especially by regular SPR detection system [28,40,41]. It allows such significant improvement by focusing on locally secreted cytokines within the immediate vicinity of the captured secreting T cells (Figure 6 and Figure 7).

## 4. Conclusions

As a conclusion, we have successfully built a biochip suitable for specifically capturing viable T lymphocytes, activating, and culturing them directly on the chip for a long incubation time (up to 24-h or more) without affecting their viability and activity, and finally detecting their cytokine secretions. The long-term culture and analysis of cells provided by this cell biochip platform allows solving one of today's major challenges on cytokine analysis: the monitoring of cytokines by identified cells. The biochip can be easily adapted to other cell types and to other secreted products to study specific biologic processes. Such analyses, in providing accurate measures of secretions and precise assessment of their sources will improve the relevance of the interpretation. This is especially critical to understand the complexity of the communications mediated by cytokines between cells where a given cytokine can be produced by several types of cells and where a given type of cell can secrete several cytokines. These considerations have raised the need of simultaneous assessment of several cytokines’ levels accordingly to the typing of the cells [42]. To this respect, our study provides improvements which were possible by taking advantage of the concept of microfluidics combined with the cell biochip format based on cell-specific and cytokine-specific antibodies. Hence, the cell biochip described here offers a number of advantages compared to current cytokine monitoring methods notably routinely used flow cytometry, ELISA, and ELISpot: (1) the cell identification and cytokine detection can be performed on-chip, in the same microdevice; (2) cytokine production is associated with specific viable cells; (4) the use of microfluidics allows minimizing sample size requirement (*i.e.*, 10^6^ cells in 100 µL of sample are used) and to control shear stress inside the device; and (5) the ease of setting up and handling of such biochip may allow its further use in routine. Additionally, the immunosandwich detection assay combined with this cell biochip platform allows having qualitative data, such as (1) evaluating secreting cells among all the T cell sub-population and/or complex biological samples; and (2) assessing the rate of apparent change (increase or decrease) in the cytokine secretion relative to fluorescence intensity detected. Furthermore, this biochip platform is highly flexible and thus suitable for multi-target detection as several probes can be arrayed in a single biochip. In addition, it has been especially designed to be compatible with both optical microscopy and SPRi; therefore providing a wide spectrum of analyses including detection of global and/or local cell secretion, and real-time and label-free detection of multiple cytokines. Its functionality could also be expanded to enable real-time and label-free detection of multiple cytokines from individual cells. SPRi is particularly suitable, because SPR phenomenon is sensitive to any optical index change within a few hundreds of nanometer above the gold layer [26,28,30,43], therefore enabling the dynamic analysis of individual cell cytokine secretions in both label-free and real-time manner. Hence, this cell biochip method will ultimately provide a very useful tool for the analysis of biomarkers and cell secreting activity and the monitoring of immune responses.

## Supplementary Files

Supplementary File 1

## References

[1] Webb J.T., Behar M. (2015). Topology, dynamics, and heterogeneity in immune signaling. Wiley Interdiscip. Rev. Syst. Biol. Med..

[2] Oppenheim J.J. (2001). Cytokines: Past, present, and future. Int. J. Hematol..

[3] Pala P., Hussell T., Openshaw P.J. (2000). Flow cytometric measurement of intracellular cytokines. J. Immunol. Methods.

[4] Ibrahim F.S., van den Engh G. (2003). High-speed cell sorting: Fundamentals and recent advances. Curr. Opin. Biotechnol..

[5] Desombere I., Meuleman P., Rigole H., Willems A., Irsch J., Leroux-Roels G. (2004). The interferon gamma secretion assay: A reliable tool to study interferon gamma production at the single cell level. J. Immunol. Methods.

[6] Leng S.X., McElhaney J.E., Walston J.D., Xie D., Fedarko N.S., Kuchel G.A. (2008). ELISA and multiplex technologies for cytokine measurement in inflammation and aging research. J. Gerontol. A Biol. Sci. Med. Sci..

[7] Trune D.R., Larrain B.E., Hausman F.A., Kempton J.B., MacArthur C.J. (2011). Simultaneous measurement of multiple ear proteins with multiplex ELISA assays. Hear. Res..

[8] Letsch A., Scheibenbogen C. (2003). Quantification and characterization of specific T-cells by antigen-specific cytokine production using ELISPOT assay or intracellular cytokine staining. Methods.

[9] Gazagne A., Claret E., Wijdenes J., Yssel H., Bousquet F., Levy E., Vielh P., Scotte F., Goupil T.L., Fridman W.H. (2003). A Fluorospot assay to detect single T lymphocytes simultaneously producing multiple cytokines. J. Immunol. Methods.

[10] Giulietti A., Overbergh L., Valckx D., Decallonne B., Bouillon R., Mathieu C. (2001). An overview of real-time quantitative PCR: Applications to quantify cytokine gene expression. Methods.

[11] Barten M.J., Rahmel A., Bocsi J., Boldt A., Garbade J., Dhein S., Mohr F.W., Gummert J.F. (2006). Cytokine analysis to predict immunosuppression. Cytometry A.

[12] Han Q., Bagheri N., Bradshaw E.M., Hafler D.A., Lauffenburger D.A., Love J.C. (2012). Polyfunctional responses by human T cells result from sequential release of cytokines. Proc. Natl. Acad. Sci. USA.

[13] Shirasaki Y., Yamagishi M., Shimura N., Hijikata A., Ohara O. (2013). Toward an understanding of immune cell sociology: Real-time monitoring of cytokine secretion at the single-cell level. IUBMB Life.

[14] Chen D.S., Soen Y., Stuge B.T., Lee P.P., Weber S.J., Brown O.P., Davis M.M. (2005). Marked Differences in Human Melanoma Antigen-Specific T Cell Responsiveness after Vaccination Using a Functional Microarray. PLoS Med..

[15] Stone J.D., Demkowicz W.E., Stern L.J. (2005). HLA-restricted epitope identification and detection of functional T cell responses by using MHC-peptide and costimulatory microarrays. Proc. Natl. Acad. Sci. USA.

[16] Chen D.S., Davis M.M. (2006). Molecular and functional analysis using live cell microarrays. Curr. Opin. Chem. Biol..

[17] Jones C.N., Lee J.Y., Zhu J., Stybayeva G., Ramanculov E., Zern M.A., Revzin A. (2008). Multifunctional Protein Microarrays for Cultivation of Cells and Immunodetection of Secreted Cellular Products. Anal. Chem..

[18] Zhu H., Stybayeva G., Macal M., Ramanculov E., George M.D., Dandekar S., Revzin A. (2008). A microdevice for multiplexed detection of T-cell-secreted cytokines. Lab Chip.

[19] Chen A., Vu T., Stybayeva G., Pan T., Revzin A. (2013). Reconfigurable microfluidics combined with antibody microarrays for enhanced detection of T-cell secreted cytokines. Biomicrofluidics.

[20] Liu Y., Yan J., Howland M.C., Kwa T., Revzin A. (2011). Micropatterned aptasensors for continuous monitoring of cytokine release from human leukocytes. Anal. Chem..

[21] Liu Y., Kwa T., Revzin A. (2012). Simultaneous detection of cell-secreted TNF-α and IFN-γ using micropatterned aptamer-modified electrodes. Biomaterials.

[22] Kwa T., Zhou Q., Gao Y., Rahimian A., Kwon L., Liu Y., Revzin A. (2014). Reconfigurable microfluidics with integrated aptasensors for monitoring intercellular communication. Lab Chip.

[23] Cherif B., Villiers L.C., Paranhos-Baccala G., Calemczuk R., Marche N.P., Livache T., Villiers M.B. (2006). Design and application of a microarray for flluorescence and SPR imaging analysis of peptide-antibody interactions. J. Biomed. Nanotechnol..

[24] Piliarik M., Homola J. (2006). SPR Sensor Instrumentation. Springer Serf. Chem. Sens. Biosens..

[25] Stybayeva G., Kairova M., Ramanculov E., Simonian A.L., Revzin A. (2010). Detecting interferon-gamma release from human CD4 T-cells using surface plasmon resonance. Colloids Surf. B Biointerfaces.

[26] Milgram S., Bombera R., Livache T., Roupioz Y. (2012). Antibody microarrays for label-free cell-based applications. Methods.

[27] Assenmacher M., Löhning M., Scheffold A., Manz R.A., Schmitz J., Radbruch A. (1998). Sequential production of IL-2, IFN-gamma and IL-10 by individual staphylococcal enterotoxin B-activated T helper lymphocytes. Eur. J. Immunol..

[28] Suraniti E., Sollier E., Calemczuk R., Livache T., Marche P.N., Villiers M.B., Roupioz Y. (2007). Real-time detection of lymphocytes binding on an antibody chip using SPR imaging. Lab Chip.

[29] Weaver W.M., Dharmaraja S., Milisavljevic V., di Carlo D. (2011). The effects of shear stress on isolated receptor-ligand interactions of Staphylococcus epidermidis and human plasma fibrinogen using molecularly patterned microfluidics. Lab Chip.

[30] Laplatine L., Leroy L., Calemczuk R., Baganizi D., Marche P.N., Roupioz Y., Livache T. (2014). Spatial resolution in prism-based surface plasmon resonance microscopy. Opt. Express.

[31] Erickson D., Sinton D., Li D. (2003). Joule heating and heat transfer in poly(dimethylsiloxane) microfluidic systems. Lab Chip.

[32] De Mello A.J., Habgood M., Llewellyn L.N., Welton T., Wootton R.C.R. (2004). Precise temperature control in microfluidic devices using Joule heating of ionic liquids. Lab Chip.

[33] Miralles V., Huerre A., Malloggi F., Jullien M.C. (2013). A Review of Heating and Temperature Control in Microfluidic Systems: Techniques and Applications. Diagnostics.

[34] Altman A., Mally M.I., Isakov N. (1992). Phorbol ester synergizes with Ca2+ ionophore in activation of protein kinase C (PKC)a and PKCfi isoenzymes in human T cells and in induction of related cellular functions. Immunology.

[35] Szmacinski H., Toshchakov V., Piao W., Lakowicz J.R. (2013). Imaging of Protein Secretion from a Single Cell Using Plasmonic Substrates. Bionanoscience.

[36] Li Y., Nath N., Reichert M.W. (2003). Parallel Comparison of Sandwich and Direct Label Assay Protocols on Cytokine Detection Protein Arrays. Anal. Chem..

[37] Kaplan D. (1996). Autocrine secretion and the physiological concentration of cytokines. Immunol. Today.

[38] Wang S., Ota S., Guo B., Ryu J., Rhodes C., Xiong Y., Kalim S., Zeng L., Chen Y., Teitell M.A. (2011). Subcellular resolution mapping of endogenous cytokine secretion by nano-plasmonic-resonator sensor array. Nano Lett..

[39] Mittal N. (2012). Cell Surface Concentrations and Concentration Ranges for Testing In Vitro Autocrine Loops and Small Molecules. PLoS ONE.

[40] Scarano S., Mascini M., Turner A.P., Minunni M. (2010). Surface plasmon resonance imaging for affinity-based biosensors. Biosens. Bioelectron..

[41] Milgram S., Cortes S., Villiers M.B., Marche P., Buhot A., Livache T., Roupioz Y. (2011). On chip real time monitoring of B-cells hybridoma secretion of immunoglobulin. Biosens. Bioelectron..

[42] Sachdeva N., Asthana D. (2007). Cytokine quantitation: Technologies and applications. Front. Biosci..

[43] Yanase Y., Hiragun T., Ishii K., Kawaguchi T., Yanase T., Kawai M., Sakamoto K., Hide M. (2014). Surface plasmon resonance for cell-based clinical diagnosis. Sensors.

