# Supplementary material for: A Simple Microfluidic Platform for Long-Term Analysis and Continuous Dual-Imaging Detection of T-Cell Secreted IFN-γ and IL-2 on Antibody-Based Biochip
Source: Biosensors (Basel). 2015 Dec 4;5(4):750–67. doi: 10.3390/bios5040750 (PMC4697143; doi:10.3390/bios5040750)
Supplement: Supplementary File 1 [file biosensors-05-00750-s001.pdf]

OPEN ACCESS

biosensors

ISSN 2079-6374

www.mdpi.com/journal/biosensors/

Supplementary Information

A Simple Microfluidic Platform for Long-Term Analysis and Continuous Dual-Imaging Detection of T-Cell Secreted IFN-γ and IL-2 on Antibody-Based Biochip. *Biosensors* 2015, *5*, 750-767

Dieudonn é R. Baganizi [1,2,3,4,5], Lo ï c Leroy [1,2,3], Lo ï c Laplatine [1,2,3], Stacie J. Fairley [1,2,3], Samuel Heidmann [1,2,3], Samia Menad [1,2,3], Thierry Livache [1,2,3], Patrice N. Marche [4,5] and Yoann Roupioz [1,2,3,*]

[1] University of Grenoble Alpes, INAC-SPRAM, Grenoble F-38000, France; E-Mails: drbaganizi@gmail.com (D.R.B.); loic.leroy@ujf-grenoble.fr (L.L.); loic.laplatine@gmail.com (L.L.); sjfairley@hotmail.com (S.J.F.); samuel.heidmann@cea.fr (S.H.); samia.menad@cea.fr (S.M.); thierry.livache@cea.fr (T.L.)

[2] CEA, INAC-SPrAM, Grenoble F-38000, France

[3] CNRS, INAC-SPrAM, Grenoble F-38000, France

[4] University Grenoble Alpes, Institut Albert Bonniot, Grenoble F-38000, France; E-Mail: marchep@ujf-grenoble.fr

[5] INSERM U823, Grenoble F-38000, France

* Author to whom correspondence should be addressed; E-Mail: Yoann.Roupioz @cea.fr; Tel.: +33-4-38-78-98-79; Fax: +33-4-38-78-51-45.

---

1. Supplemental Information I

1.1. Experimental Temperature Calibration

The temperature inside the chamber (=flowcell) is estimated based on an experimental calibration $T_{flowcell} = f(T_{ITO})$. This calibration is performed by inserting and binding with a UV curable glue a second thermistor into a drilled prism, so that its head goes just above the prism base (~100 µm), and is in the middle of the flowcell. Water is inserted in the fluidic device and both temperatures are monitored simultaneously with a homemade LabVIEW program (Figure S1).

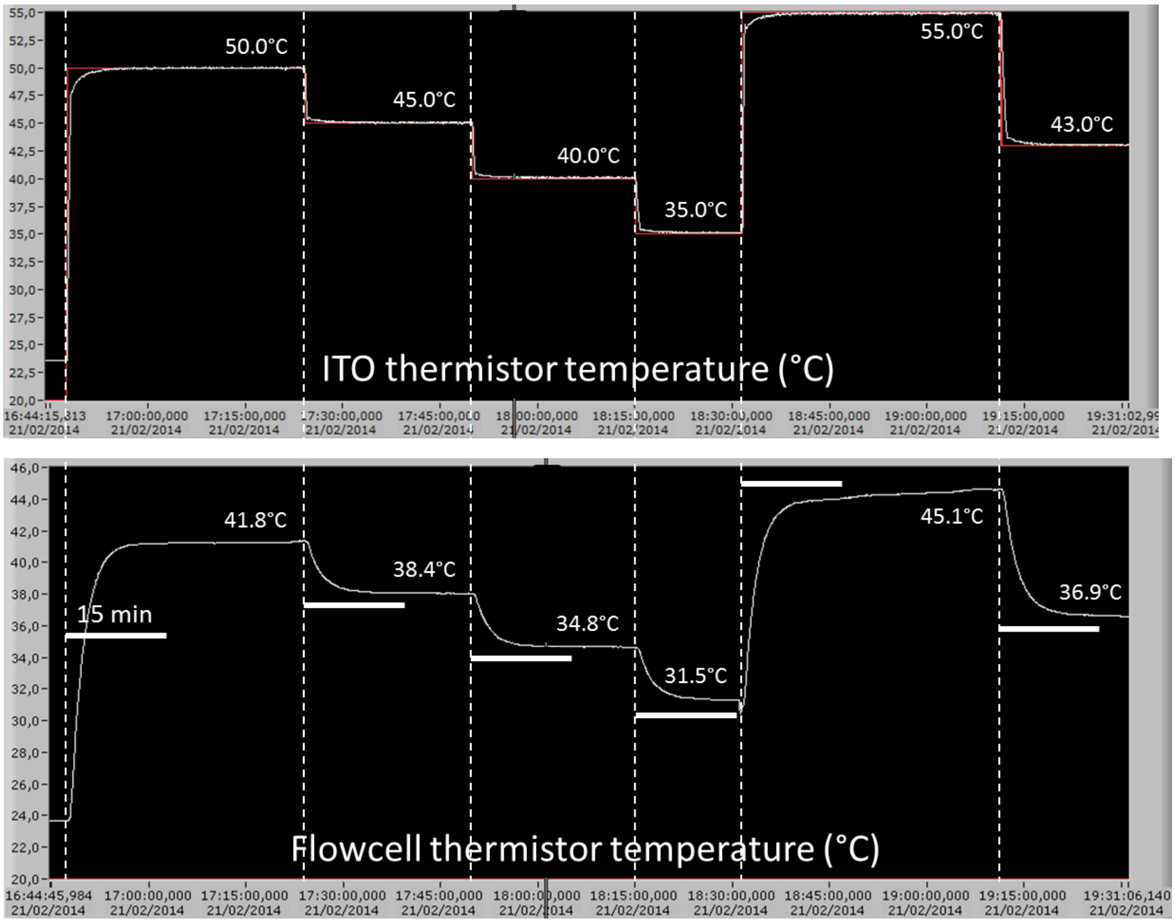

Figure S1. Simultaneous measurements of the ITO and flowcell temperatures.

While T_{ITO} stabilizes in only a few seconds, $T_{flowcell}$ needs ~10 min to asymptotically stabilize due to the thermal inertia of the system. However, Figure S2 shows a very linear relation between the two over the range of desired temperatures at equilibrium.

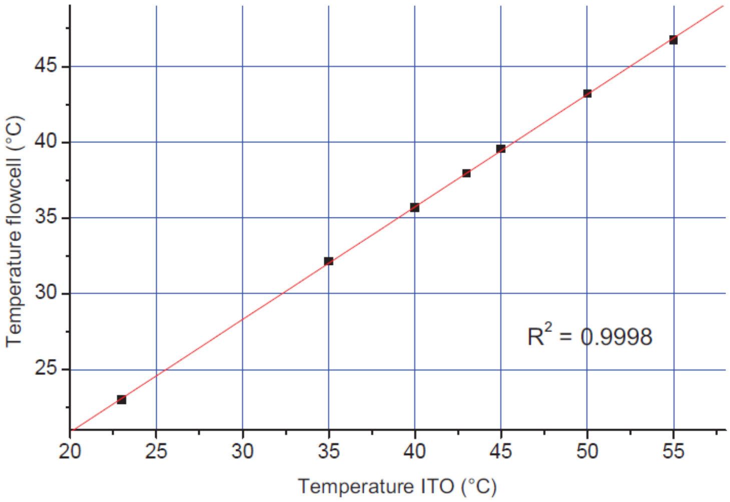

Figure S2. Experimental temperature calibration between the flowcell and the ITO layer.

Furthermore, to test the stability of the technique, both temperatures were monitored over more than 14 hours (Figure S3). Note that this experiment has been performed on a previous version of the current design were the prism was not entirely drill and the thermistor head was located approximately 2–3 mm below the sensing surface, therefore T_{ITO} was set at 50 °C to have T_{prism} at 37 °C.

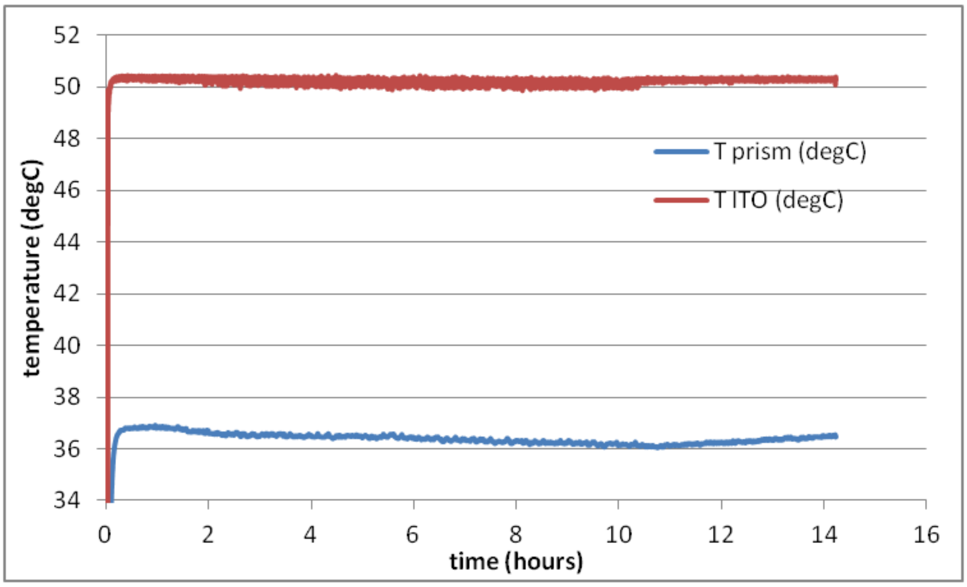

Figure S3. Temperature stability of the regulation system.

<u>Simulation:</u>

The thermal conductivity of PDMS were taken from Ref [1] and is k = 0.2 W/(m·K). The thermal conductivity of the glass support and the ITO coated glass slide were the one provided by the SolidWorks material library for fused silicate glass with k = 1.38 W/(m·K). The thermal conductivity of the N-SF66 prism was taken from the Schott datasheet and is k = 0.800 W/(m·K). The thermal conductivity of water is k = 0.609 W/(m·K) (SolidWorks material library)

ITO coated glass slide is 1.1 mm thick. The glass support is 3 mm thick. The 1-mm-thick PDMS layer is a rectangle with the same dimension as the prism base (12.5 × 25 mm). It has a central opening region (as visible on Figure 4C-2 in the manuscript) containing water. The prism has an apex angle of 83 °C.

The limit conditions for the temperature are 42 °C on the ITO surface and 23 °C at the edges of the principal glass plate and at the two prism sides (not faces) which are in contact with the prism support, as indicated by the arrows on Figure 4C-1 in the manuscript. Figure 4C-2 in the manuscript shows the temperature at the prism base. Even if these limit conditions are overestimated (the prism sides and the principal glass plate edges are surely heated up above the ambient temperature), this simulation gives results close to the measurements: 37 °C at the center of the flowcell, as measured in Figure S2.

A part of the heat is dissipated by convection in the ambient air. Most of horizontal surfaces have a convection heat transfer coefficient in air of ~10 $W.m^{-2}.K^{-1}$ [2], which leads to a dissipation in the order of 0.2 W for our system. Considering the total power consumption measured at 1.9 W at equilibrium, heat transfer by convection and radiation were neglected in the simulation.

The temperature of the flowcell $T_{flowcell}$ during an SPR experiment cannot be measured directly because of its small dimensions. It is more practical to measure and regulate the temperature of the ITO plate T_{ITO}, and correlate it to $T_{flowcell}$ by a calibration. T_{ITO} is monitored by a NTC thermistor (Negative Temperature Coefficient) which resistance RT exponentially decreases when temperature increases. RT is calculated by a LabVIEW program by simultaneously measuring the voltage V_{ref} of a reference resistor of a known resistance ($R_{ref} \approx 10$ k) and the voltage of the thermistor V_T by a LabJack acquisition card (Figure 4B in the manuscript). In our electric set-up, $R_T = R_{ref}(V_T/V_{ref})$. To establish the function $R_T = f(T)$, the thermistor is immersed in a glass of water which temperature decreases from 83 °C to 23 °C (measured with a reference commercial thermometer) and R_T is measured every 5 °C. This range makes R_T vary from 500 to 6000 ohms. The measuring points are fitted by an exponential decaying function. T_{ITO} is finally monitored by placing the thermistor head in contact with the ITO layer and by adding some thermal paste to ensure a good thermal contact.

2. Supplemental Information II

2.1. Peripheral Blood T Lymphocytes: Activation and IFN-γ and IL-2 Secretion Analyses

The secretory activity of IFN-γ and IL-2 by PMA/Ionomycin activated peripheral blood T lymphocytes over time was evaluated by ELISA assay in cell supernatant collected at different incubation times under optimum culture condition. Cells were incubated at 1×10^6 cells/mL in a 24-well plate at 1×10^6 cells/mL in 1 mL of AIM-V Serum Free Medium buffered with 25 mM Hepes (GIBCO), in the presence or not of T cell activating agents (100 ng/ml PMA associated with 500 ng/mL of Ionomycin). The supernatant was then collected after 6 h, 12 h, and 24 h of incubation. A native well (without the activating agent) of cells from the same sample was incubated under the same conditions as the negative control. The ELISA assay was performed using eBiosciences Human ELISA IFN-γ or IL-2 Ready-Set-Go (10 × 96 Tests) protocol kit, and following the kit instructions. Briefly, the plates (96-well ELISA plates, Falcon™ BD) were coated with anti-IL-2 or anti-IFN-γ capture antibody overnight at 4 °C, washed and blocked with specific buffers of the kit. Then samples and standards were added to the wells. Bound antibodies were revealed with streptavidin-HRP using tetramethyl benzidine as the substrate (TMB). Standard curves were plotted using dilutions of the standard solution provided in the kit.

The ELISA assay analysis of IL-2 and IFN-γ of peripheral blood T lymphocytes cellular supernatants indicates that PMA/Ionomycin activation of peripheral blood T lymphocytes results in high concentrations of secreted IL-2 and IFN-γ compared to unstimulated cells which secret very low amounts of IL-2 and IFN-γ or none at all (Figure S4).

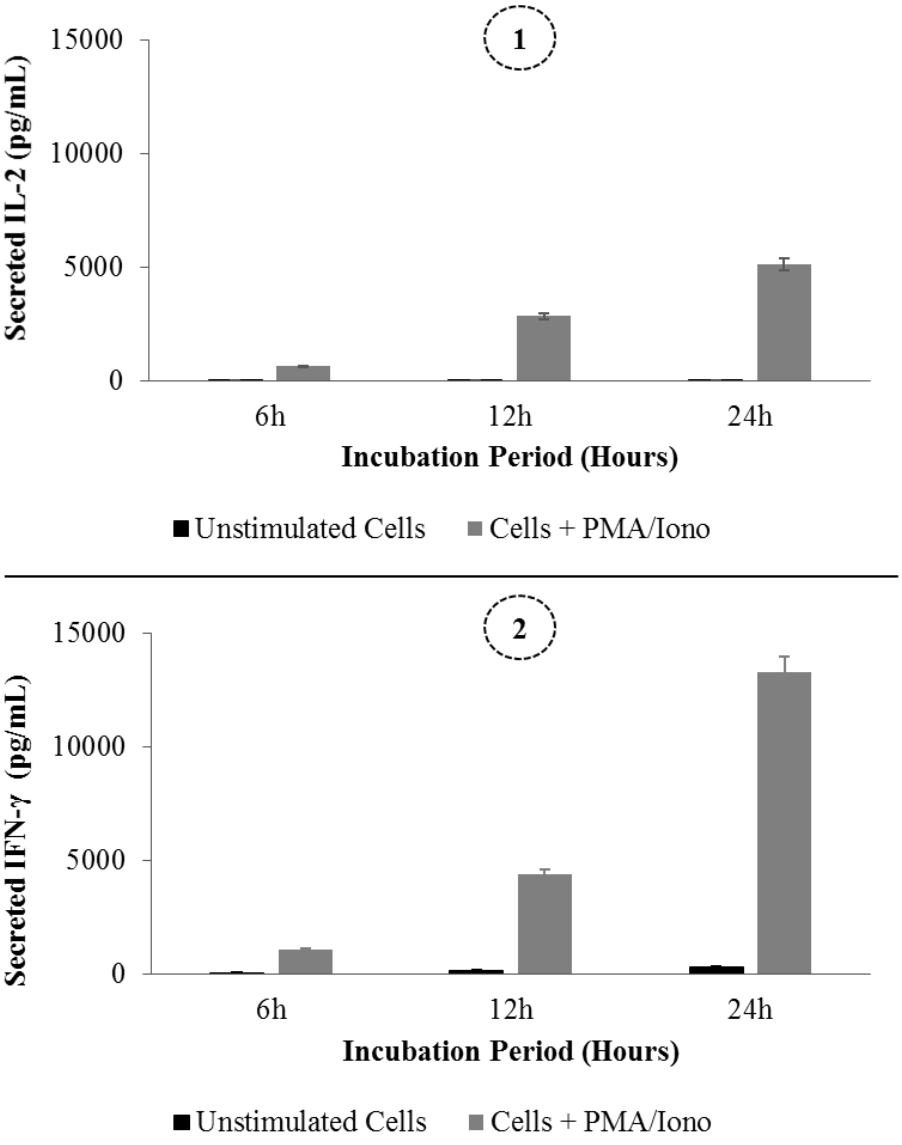

Figure S4. ELISA titration of IL-2 (1) and IFN-γ (2) contained in peripheral blood T-lymphocyte cellular supernatants after cell culture in 24-well plate at 1×10^6 cells/mL.

3. Supplemental Information III

3.1. SPRi Detection Curves over Time of Captured T Cell Cytokine Secretions before Rectification by the Subtraction of the Control Signals

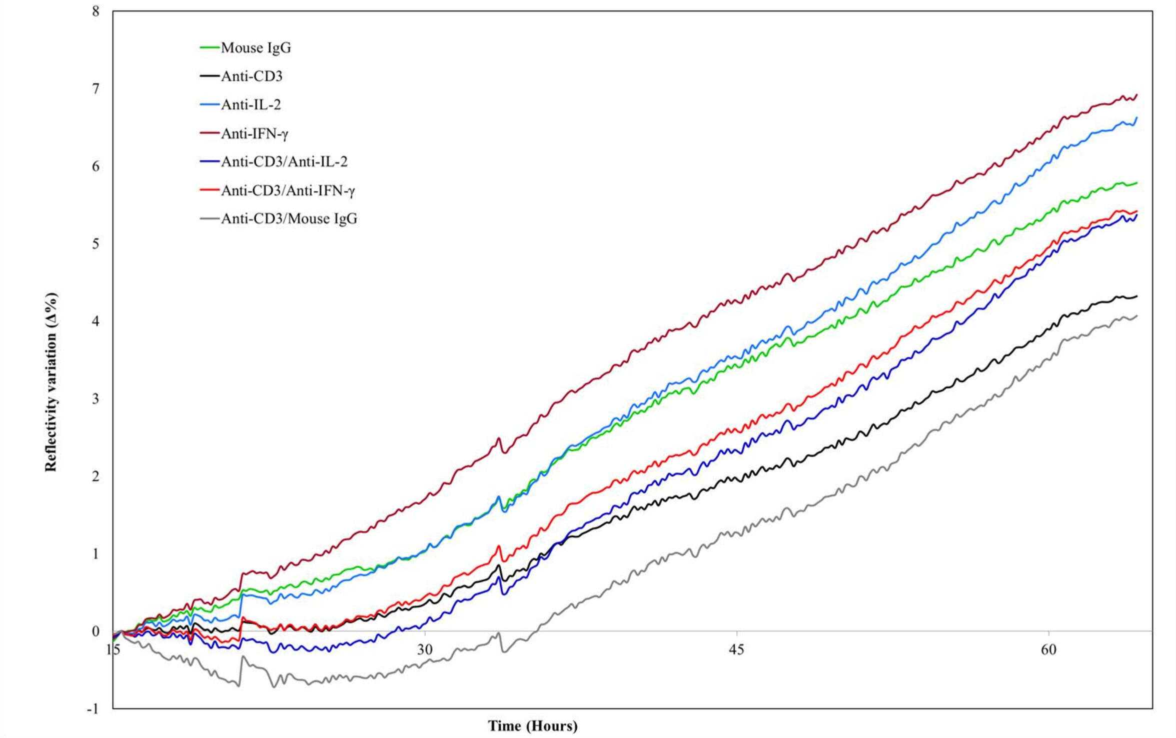

Figure S5. Continuous SPRi dual-detection of IFN-γ and IL-2 secreted by captured peripheral blood T lymphocytes. After cell injection in the microfluidic chamber and capture on their specific antibody probes (anti-CD3, mixture of anti-CD3/anti-IL-2 and anti-CD3/anti-IFN-γ), secreted cytokines are continuously detected by SPRi. Averaged reflectivity variations of each probe plotted over time before rectification by the subtraction of the control signals (mouse IgG and/or anti-CD3 mixed with mouse IgG).

References

1. Tang, S.; Whitesides, G. Basic microfluidic and soft lithographic techniques. In *Optofluidics Fundamentals, Devices, and Applications*; McGraw-Hill Professional: New York, NY, USA, 2010; Chapter 2.
2. Krdgerl, D.G. Convection heat transfer between a horizontal surface and the natural environment. *RandD J.* **2002**, *18*, 49–54.

© 2015 by the authors; licensee MDPI, Basel, Switzerland. This article is an open access article distributed under the terms and conditions of the Creative Commons Attribution license (http://creativecommons.org/licenses/by/4.0/).
